# Prevalence and Evolutionary Implications of Genome Rearrangements in Bacteria

**DOI:** 10.1093/gbe/evag002

**Published:** 2026-01-23

**Authors:** Carolina A Martinez-Gutierrez, Yuqi Huang, Louis-Marie Bobay

**Affiliations:** Department of Biological Sciences, North Carolina State University, Raleigh, USA; Bioinformatic Research Center, North Carolina State University, Raleigh, USA; Department of Earth Science, University of California, Santa Barbara, USA; Department of Biological Sciences, North Carolina State University, Raleigh, USA; Bioinformatic Research Center, North Carolina State University, Raleigh, USA; Department of Biological Sciences, North Carolina State University, Raleigh, USA; Bioinformatic Research Center, North Carolina State University, Raleigh, USA

**Keywords:** genome rearrangement, synteny, transposable elements, genome rearrangement rates, origin of replication

## Abstract

The genetic material of bacteria and archaea is organized into various structures and setups, attesting that genome architecture is dynamic in these organisms. However, strong selective pressures are also acting to preserve genome organization, and it remains unclear how frequently genomes experience rearrangements and what mechanisms lead to these processes. Here, we assessed the dynamics and the drivers of genomic rearrangements across 121 microbial species. We show that synteny is highly conserved within most species, although several species present exceptionally flexible genomic layouts. Our results show that genomic rearrangements occur at a variable pace across bacteria and archaea, pointing to different selective constraints driving the accumulation of genomic changes across species. Importantly, we found that not only inversions but also translocations are highly enriched near the origin of replication (*Ori*), which suggests that many rearrangements may confer an adaptive advantage to the cell through the relocation of genes that benefit from gene dosage effects. Finally, our results confirm the view that mobile genetic elements—in particular transposable elements—are the main drivers of genomic translocations and inversions. Overall, our study shows that microbial species present largely stable genomic layouts and identifies key patterns and drivers of genome rearrangements in prokaryotes.

Significance Although microbial genomes are frequently reorganized over long evolutionary timespans, the dynamic of genome rearrangements is less clear over shorter timescales. By analyzing the genomes of 121 microbial species, we show that genome organization is remarkably stable within species, with a single rearrangement event per genome on average. However, a minority of genomes exhibited extensive rearrangements indicating that genome architecture can evolve rapidly. Our findings suggest that most genome rearrangements are rapidly purged by selection. We also identified a sharp increase of translocations and inversions near the origin of replication. Our results further support the view that mobile genetic elements—particularly transposable elements—play a central role in driving these events. This work refines our understanding of the dynamics of genome architecture in microbes by characterizing the patterns and the pace of genomic rearrangements across microbial lineages.

## Introduction

Bacterial genomes are highly organized entities that evolve under the selection pressures imposed by functional constraints such as replication, transcription, DNA repair, and cell division (Casjens 1998). Over long timescales, genomes have evolved into diverse chromosomal setups (e.g. circular/linear chromosomes, single/multiple chromosomes) through processes mediated by homologous recombination, horizontal gene transfer, and mobile genetic elements (MGEs) (Casjens 1998; Rocha 2008; Darmon and Leach 2014). Multiple studies have shown that genomes are not simply the set of genes of an organism (Rocha 2004, 2006, 2008; Junier and Rivoire 2013). Instead, genomes display a complex architecture whose organization is shaped and constrained by natural selection to facilitate cell processes (Casjens 1998; Rocha 2008). For instance, genes involved in the same function are often organized into operons, and essential genes that are highly expressed tend to be found on the leading strand and in close proximity to the origin of replication (*Ori*) (Romero and Palacios 1997; Rocha 2008). Genes located near *Ori* are highly expressed because this DNA region is present in two or more copies when the chromosome is actively replicating (i.e. gene dosage effect) (Schmid and Roth 1987; Sousa et al. 1997; Rocha 2008). In addition, microbial genomes harbor polarized short DNA motifs that play essential roles for chromosome stability and duplication (Rocha 2008). For instance, *Chi* sites are eight-nucleotide-long motifs present on the leading strand of Enterobacteria, where they play a key role in DNA repair (Stahl 2005; Buton and Bobay 2021). In *Escherichia coli*, polarized motifs such as *KOPS* are found near the terminus of replication (*Ter*) and are essential for the proper segregation of the two chromosomes during the termination of DNA replication (Rebollo et al. 1988; Rocha 2008; Nolivos et al. 2012). In *E. coli,* the chromosome is structured into four macrodomains and two unstructured regions (Valens et al. 2004). This architecture is important for chromosome dynamics during the cell cycle and the shuffling of macrodomains and the motifs they contain is expected to deleteriously affect cell fitness (Valens et al. 2004; Esnault et al. 2007).

Due to the essential role of genome organization in bacteria, genomic rearrangements are thought to be rare because they often alter the architecture and polarity of the genome (Rocha 2008). For instance, genome inversions are expected to deleteriously affect cell fitness by reversing the polarized DNA motifs along the chromosome(s) (Esnault et al. 2007). Other studies have suggested that—despite the constraints of chromosome architecture—certain types of rearrangements can be rather common in bacteria. It has been shown that *E. coli*, *Haemophilus influenzae*, and *Helicobacter pylori* undergo rearrangements due to recombination, but inversions tend to be symmetrical relative to the replication axis, which preserves strand polarity (Tillier and Collins 2000). However, studies focusing on the impact of genomic rearrangements on cellular fitness have led to contrasting conclusions. Several works have shown that genomic inversions can play a positive role in the ability of microbes to evade host defenses (Kresse et al. 2003; Rocha 2004; Merrikh and Merrikh 2018) and adapt to new niches (Repar et al. 2017; Cao et al. 2022), but other studies demonstrated that these events lead to lower growth rates and even cell death (Campo et al. 2004; Rocha 2004).

Current knowledge on genomic rearrangements is based on a narrow set of microbial clades or by comparing relatively old events through the comparison of different species to one another (Rocha 2006; Brilli et al. 2013). It remains unclear how frequently these events occur within species, what processes drive their occurrence, and to what extent they can impose deleterious, neutral, or beneficial selective pressures. In this study, we explore the prevalence and the potential impacts and drivers of genome rearrangements across a broad diversity of microbial species. By focusing our analyses on a large set of high-quality assemblies, we show that genomic rearrangements remain rare in the vast majority of the species analyzed, although their rates vary greatly across species. We found evidence that selection purges most of the rearrangements occurring in microbial genomes. Most rearrangements were found to occur near and symmetrically to *Ori*, suggesting that these events are less deleterious—or potentially beneficial—when occurring in this region. Interestingly, rearrangements near *Ori* are not restricted to inversions, but translocations are also enriched in this region. These results suggest that rearrangements are not predominantly composed of inversions occurring near *Ori,* which are thought to be less detrimental to the cell by preserving strand polarity. Rather, these patterns support the view that many rearrangements may be adaptive through the relocation of genes near *Ori* to benefit from increased expression through gene dosage effect. Finally, we present evidence showing that transposable elements and other MGEs are likely the main drivers of genome rearrangements in bacterial and archaeal species.

## Results

### Identification of Genomic Rearrangements in Bacterial and Archaeal Species

In order to explore the prevalence of genome rearrangements across a broad diversity of prokaryotic species, we collected high-quality genomes (100% complete and <5% contaminated) belonging to archaea and bacteria from the Genome Taxonomy Database v207 (GTDB; GTDB dataset) (Chaumeil et al. 2019). Our GTDB dataset consisted of 52,543 genomes belonging to 5,077 and 142 bacterial and archaeal species, respectively (Table S1). Given the crucial role of assembly quality on genomic rearrangement identification, we obtained the assembly information for each genome from the National Center for Biotechnology Information (NCBI) (Sayers et al. 2022). We selected the genomes that were fully assembled for further analyses, and species with fewer than five genomes were excluded. Our final dataset (Table S2) was composed of 5,238 genomes belonging to 119 bacterial species and two archaeal species.

For each species, we built the set of core genes (i.e. orthologous genes that are shared amongst most genomes of the species) using *CoreCruncher* (Harris et al. 2021), which is robust to the presence of paralogs and xenologs (see Materials and Methods). We then identified blocks of adjacent genes by comparing the order of core genes between genome pairs of the same species. We defined gene blocks as sets of consecutive and uninterrupted core genes between each genome pair, and only gene blocks composed of at least two core genes were considered to avoid biases due to the potential misassignment of gene orthology (see Materials and Methods). For each species, we selected one reference genome which was defined as the genome that led to the lowest average number of blocks in the species (Fig. S1). The number of gene blocks between genome pairs was then used to estimate the number of genome rearrangements that have occurred within each species.

To avoid overestimates in the number of gene blocks due to incorrect assemblies, we assessed the impact of sequencing technology on gene block identification. Genomes generated through short-read sequencing (e.g. Illumina) can contain assembly errors as they are more complex to assemble relative to genomes generated from long-read sequencing technologies (e.g. PacBio and Oxford NanoPore). However, we found that both sequencing technologies lead to similar numbers of blocks (Wilcoxon test, *P* = 0.44, Fig. S2). This finding indicates that short-read sequencing technologies do not appear to introduce more assembly errors than long-read sequencing technologies. Therefore, we used genomes generated by both, short- and long-read sequencing technologies for further analyses.

**Fig. 1.**
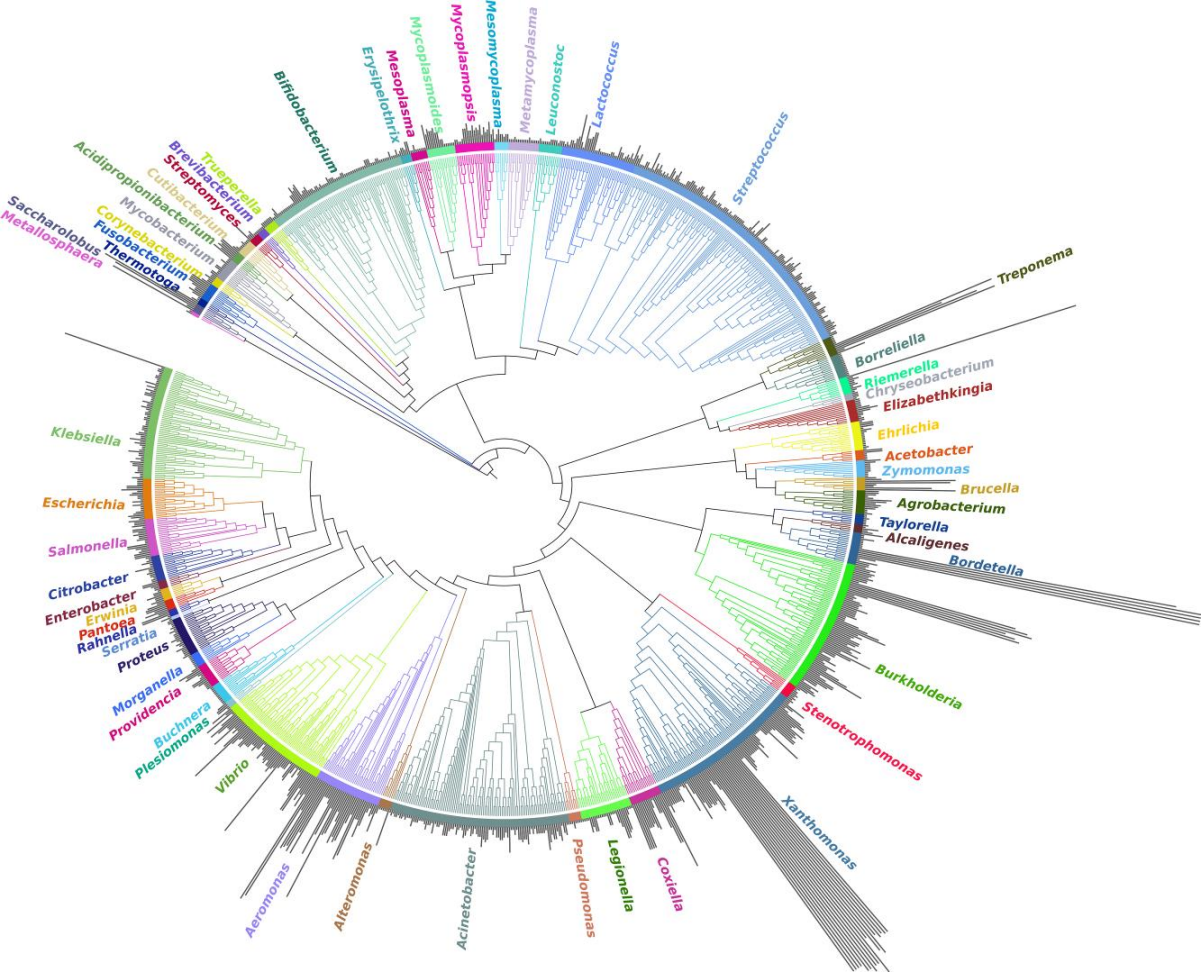
Phylogenetic breadth of the genome dataset used to estimate rearrangement dynamics. The maximum-likelihood phylogeny was constructed using 30 conserved marker genes encoding ribosomal proteins and RNA polymerase subunits. The bars adjacent to each genome represent the number of gene blocks identified, ranging from a minimum of 1 block (no rearrangements) to a maximum of 132 blocks.

**Fig. 2.**
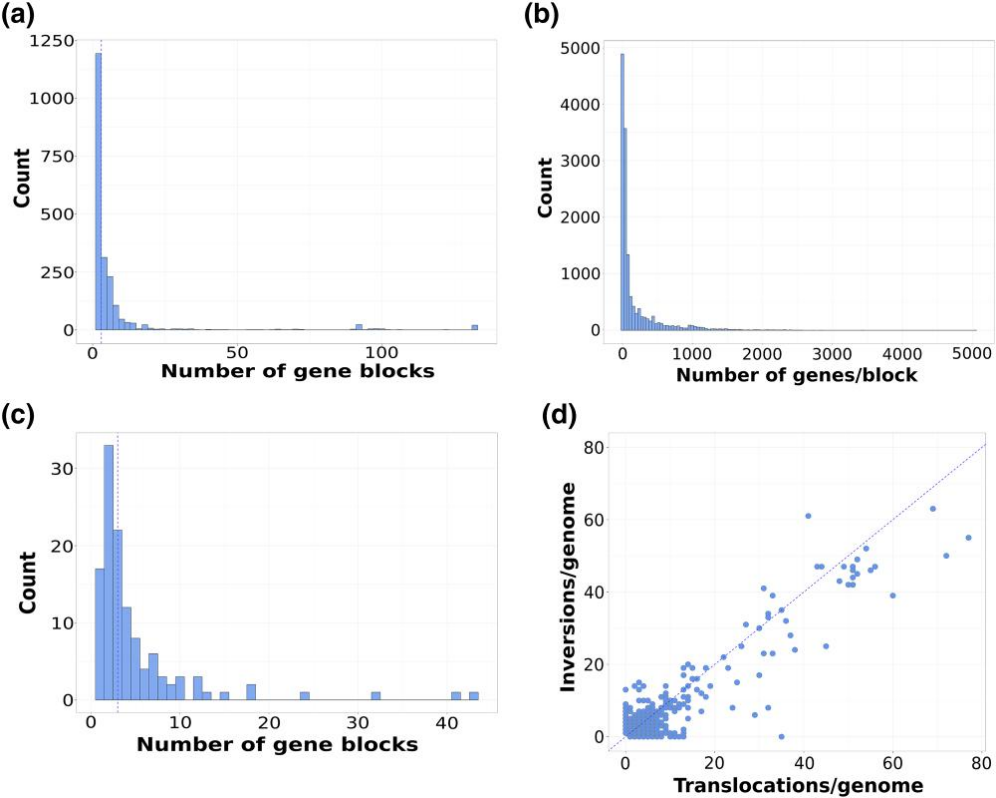
Distribution of the number of rearranged gene blocks across bacterial and archaeal species. a) Number of blocks per genome; b) length in number of genes of rearranged blocks including both accessory and core genes; c) average number of blocks per species; and d) number of inversions and translocations per genome.

We analyzed the 119 bacterial species and 2 archaeal species of our dataset, which spans a wide phylogenetic breadth of 58 genera (Fig. 1). Overall, most of the bacterial and archaeal genomes analyzed harbored few gene blocks, indicating that the reorganization of the genome structure is infrequent within species (Figs. 1 and 2a; Table S3). We found a median of three gene blocks per genome, which indicates that a genome typically displays a single rearrangement event (i.e. one rearrangement event can lead to two or three blocks, depending on the nature of the rearrangement). However, some genomes were exceptionally fragmented with up to 132 blocks (Fig. 2a; Table S3). Given the surprisingly large number of blocks found in some genomes, we questioned whether the high number of blocks in these genomes could be the result of assembly errors. However, after careful analysis, we did not find any evidence supporting this claim: (i) almost all genomes (>92%) composed of ≥20 syntenic blocks were generated using long-read assemblies or long-read and short-read hybrid assemblies (Table S4). (ii) Many of the genomes with large block numbers displayed identical synteny relative to one another, For instance, some genomes of *Bordetella pertussis* and *Xanthomonas oryzae* are composed of 132 blocks and 101 blocks, respectively, relative to the reference genomes. However, these highly fragmented genomes are almost perfectly syntenic relative to one another in both species (Fig. S5). Because these genomes were assembled de novo, independently from one another, these highly rearranged genomes are not the result of erroneous assemblies. (iii) Some of these genomes appear to have recently experienced profound rearrangements as a result to a recent burst of Insertion Sequence elements. For instance, the strain 20190502E1-H1 of the bird pathogen *Riemerella anatipestifer* is split into 91 blocks while the 11 other strains of this species are highly syntenic (organized into one to four blocks). The genome of the highly rearranged strain contains 118 genes annotated as IS982-like transposases, whereas most other strains contain fewer than seven. These different lines of evidence led us to believe that the highly fragmented genomes in our dataset are truly the result of rearrangements and not assembly errors.

The diverse species in our dataset display various genome sizes, and this can partially explain the variation in the number of rearrangements across taxa. Specifically, we examined whether differences in core genome size were associated with the number of inferred blocks. We found a large variation in core genome size across the analyzed species (Fig. S3a), and it was indeed positively correlated with the average number of blocks identified (Spearman's *ρ* = 0.33, *P* < 0.001; Fig. S3b). Core genome size, therefore, partially explains the number of blocks detected in each species. In contrast, we did not find a correlation between the number of genomes within each species and the average number of blocks (Spearman's *ρ* =−0.02, *P* = 0.80; Fig. S4a), but the number of analyzed genomes showed a weak correlation with block length (Spearman's *ρ* = −0.27, *P* = 0.002) driven by small genome sets (Fig. S4b).

A large fraction of the genomes in our dataset (32% of the total genomes analyzed) had only one gene block per genome, which indicates a complete absence of genomic rearrangements in these genomes (Fig. 2a; Table S3). We defined as “rearranged blocks” the *n*−1 smallest blocks of a genome that were considered as having been reorganized relative to the largest *n*^th^ block of the genome. On average, the rearranged blocks in our dataset were composed of 184 genes (including accessory and core genes) and few rearranged blocks exhibited more than 1,500 genes (Fig. 2b). At the species level, most taxa displayed less than five blocks on average (75% of the species considered), with several species having an average of up to 43 blocks (Fig. 2c; Table S5). We found seven species that did not exhibit any genomic rearrangements across all their genomes. These species were *Buchnera aphidicola, Treponema pallidum, Morganella morganii, Brevibacterium aurantiacum, Borreliela garinii, Borreliela burgdorferi,* and *Bordetella avium.* At the other end of the spectrum, *Aeromonas salmonicida, Bor. pertussis, Treponema phagedenis,* and *X. oryzae* had the highest number of gene blocks (>20 blocks) (Fig. 2c; Fig. S5; Table S4), which reflects a higher occurrence of rearrangements in these species, as previously reported (Tanaka et al. 2012; Weigand et al. 2017; Kaur et al. 2020). Interestingly, the number of rearrangements often differed markedly within species and between closely related species (e.g. *T*. *pallidum* vs. *T. phagedenis* and *Bor. pertussis* vs. *Bordetella holmesii,* Fig. 1). Indeed, the phylogenetic signal of block number measured across our species tree shows that evolutionary relationships had negligible predictive power on this trait (Blomberg's *K* < 0.001; Fig. 1), which indicates that rearrangements are primarily driven by species-specific factors.

Although archaeal species were included in the original dataset, only two archaeal species—*Metallosphaera sedula* and *Saccharolobus solfataricus*—remained after quality filtering. This limited representation prevents us from drawing solid conclusions about the dynamics of genomic rearrangements in archaea. Nevertheless, *M. sedula* was consistent with most bacterial species with a low average number of gene blocks (1.7 blocks per genome), whereas *S. solfataricus* displayed a markedly higher average (15 gene blocks per genome) (Tables S3 and S5).

We further tested whether the number of predicted blocks per species were potentially driven by the presence of divergent—and potentially misclassified—strains. We observed a significant correlation between the number of blocks and the Average Nucleotide Identity of genome pairs (Spearman's *ρ* = −0.12, *P* < 0.001), but this effect was weak (Fig. S6a). Moreover, the genomes presenting the most rearrangements were not particularly divergent from one another (ANI > 95%) and did not show any evidence of taxonomic misclassification (Fig. S6b). The same trend was found when assessing the potential impact of evolutionary distance between genomes and their reference (Spearman's *ρ* = −0.036, *P* = 0.3; Fig. S7). These results indicate that the levels of genomic divergence within species play a minimal role in our estimates of rearrangements, and that strain misclassification is not driving our predictions.

We next investigated the relative prevalence of translocations and inversions across the rearranged genomes (Fig. 2d). Genome translocations are characterized by relocations of a blocks of genes without a disruption of their orientation (Tillier and Collins 2000; Belda et al. 2005), whereas inversions are changes where the strand orientation of a contiguous block of genes is reversed (Eisen et al. 2000). Since the net effect of inversions is a change in the orientation of genes from the leading to the lagging strand, these rearrangements are considered to be particularly deleterious (Darling et al. 2008). However, our results show that translocations occur at a similar frequency to that of inversions, with an average of four translocations compared to three inversions across the dataset (Fig. 2d).

### Location of Translocations and Inversions Across Genomes

Previous studies based on a limited set of bacterial species suggested that genome inversions may be prevalently symmetric relative to *Ori* and *Ter* (Eisen et al. 2000; Mackiewicz et al. 2001; Rocha 2008; Kang et al. 2014). Symmetric inversions are expected to be less detrimental because they maintain the polarity of the genes in the chromosome (Rocha 2004, 2008; Darling et al. 2008). Moreover, DNA motifs are often polarized relative to the replication fork, and asymmetric inversions that disrupt their polarity relative to *Ori* or *Ter* can be deleterious (Hill and Gray 1988; Mackiewicz et al. 2001; Rocha 2004, 2008; Darling et al. 2008). To investigate whether symmetric inversions are widespread among the species analyzed, we identified the location of the inverted gene blocks relative to *Ori* and *Ter,* whose location was determined by computing the GC skew of each genome (See Materials and Methods). For this analysis, we excluded species known for having multiple or linear chromosomes (e.g. *Vibrio* and *Burkholderia*). We further excluded a species when the identification of *Ori* and *Ter* was ambiguous based on their GC-skew plots (see Materials and Methods).

**Fig. 3.**
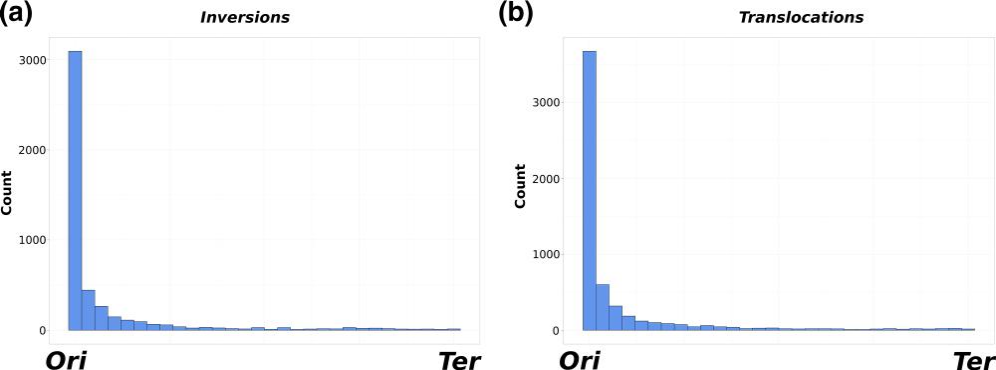
Location of genome rearrangements relative to the origin (*Ori*) and terminus (*Ter*) of replication. a) Distribution of the midpoint positions of inversion events across both chromosomal replichores and b) distribution of the midpoint positions of translocation events across both chromosomal replichores.

Consistent with previous studies, we found that inversions are preferentially located near *Ori,* and to a lesser extent *Ter* (Fig. 3a). We next tested whether this trend could be driven by the random occurrence of inversions near *Ori* by statistically comparing the distribution of the relative position of inversions with the expectation under neutrality (See Materials and Methods). Our results show that inversions are prevalently located near *Ori* when compared with the random expectation (Wilcoxon signed rank test, *P* < 0.001). Surprisingly, the same trend was found for translocations (Wilcoxon signed rank test, *P* < 0.001) (Fig. 3b), indicating that *Ori* is a hotspot for genomic rearrangements not only for inversions, but also for translocations. This result suggests that rearrangements do not only occur near *Ori* because they preserve strand symmetry since this would only explain the higher prevalence of inversions near *Ori*, but not the higher prevalence of translocations.

### Estimation of the Rate of Genome Rearrangement Across Species

**Fig. 4.**
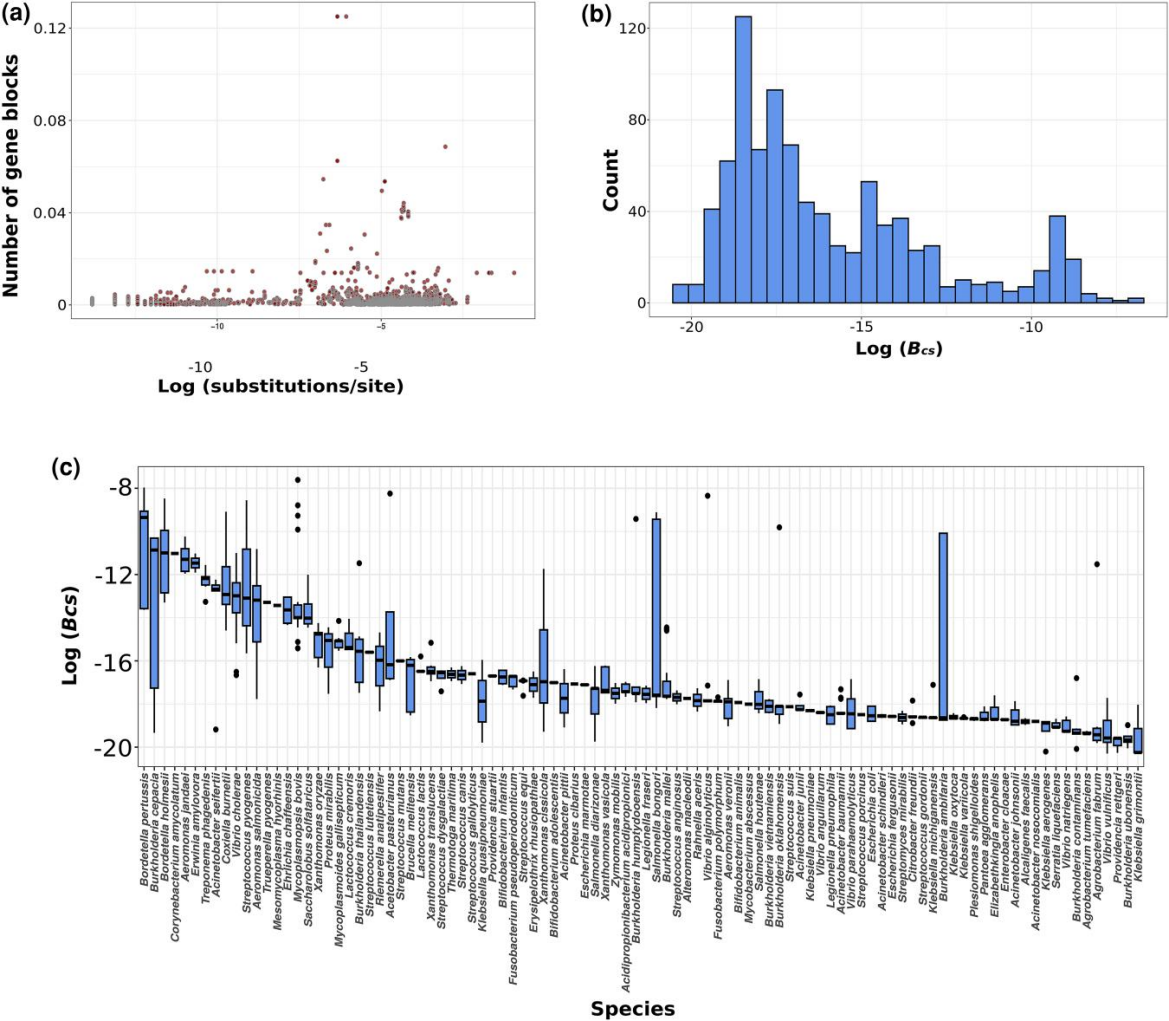
Relationship between the number of gene blocks and evolutionary distance. a) Number of gene blocks adjusted by core genome size plotted against pairwise evolutionary distance (substitutions per site) estimated from core genome alignments and b) rearrangement rates (*B_sc_*) across bacterial and archaeal genomes. *B_sc_* represents the number of estimated rearrangements divided by the product of the size of the core genome (number of genes), the length of the core genome alignment (in nucleotides), relative to the number of substitutions between genome pairs (i.e. number of blocks adjusted by the size of the core genome relative to the substitution rate). See the Materials and Methods section for more details on the estimation of *B_sc_*. c) Distribution of rearrangement rates (*B_sc_*) across species. Overall, species with a larger number of genomes showed a wider variation in rates (Fig. S8).

Earlier studies have shown that, in general, closely related lineages show more conserved synteny than distantly related ones, and that rates of rearrangements can be estimated relative to substitution rates (Suyama and Bork 2001; Belda et al. 2005; Rocha 2006; Brilli et al. 2013). Here, we tested to what extent phylogenetic distance may be a predictor of the number of genomic rearrangements (as approximated through the number of gene blocks) between closely related strains. We built a concatenate of core-genome alignments for each species and estimated the divergence between the pairs of genomes and their number of rearrangements relative to one another. In contrast to previous studies that mostly focused on divergent species (Suyama and Bork 2001; Belda et al. 2005; Rocha 2006; Brilli et al. 2013), we observed a significant but weak correlation between the evolutionary distance and the pairwise number of blocks after adjusting for core genome size (Fig. 4a; Spearman's *Rho* = 0.08; *P* < 0.001). This finding indicates that rearrangements do not accumulate at a constant pace over short evolutionary timescales (i.e. between strains of a species).

We further aimed at measuring at which rate genome rearrangements occur across the species of our dataset. We estimated the number of rearrangements from the number of blocks observed between genome pairs. To do so, we used simulations to establish a relationship between the number of rearrangements and the number of expected blocks resulting from these events (See Materials and Methods). Because the size of the core genome varies substantially across species, we defined the rate of rearrangement as *B_sc_,* which represents the number of blocks adjusted by the size of the core genome relative to the substitution rate, which allows us to compare the rate of rearrangement across species with different core genome sizes. In support of our findings of an unsteady pace of rearrangement across genomes and species, our results show a large variation of rates across genomes (Fig. 4b), but also between and within species (Fig. 4c). We observed a negative relationship between genome size and the rate of rearrangements (Spearman's *ρ =* −0.33, *P* < 0.001; Fig. S9b), indicating that species with smaller genomes undergo higher rearrangement rates as measured by the proportion of rearranged genes.

### Mobile Elements Are Likely Drivers of Genome Rearrangements

**Fig. 5.**
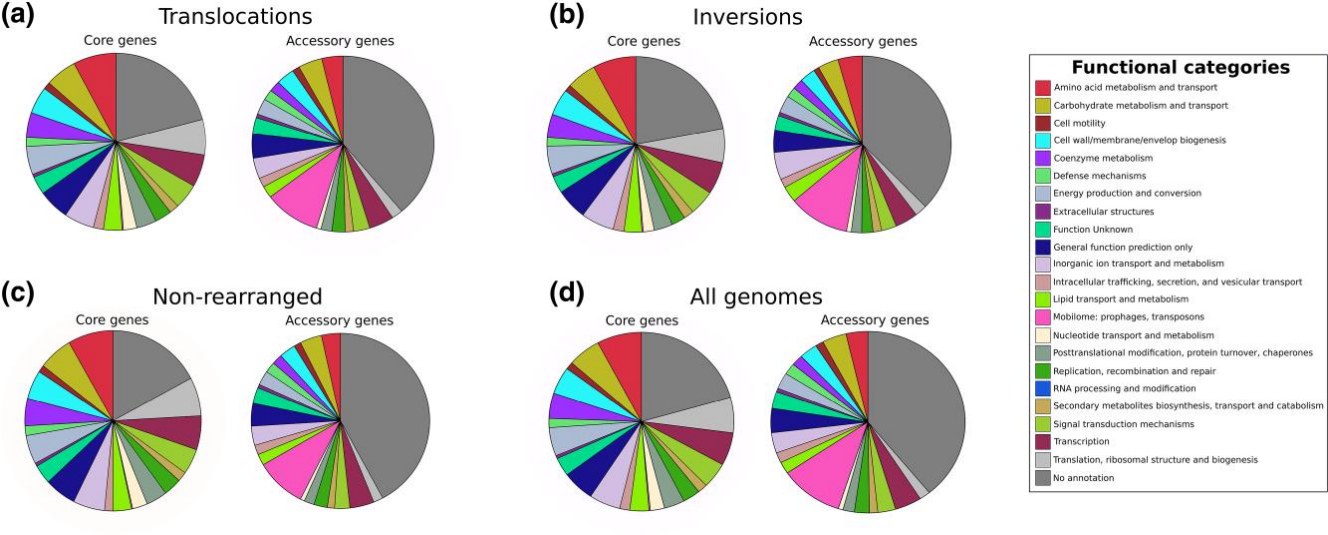
Occurrence of functional categories (COG annotations) of core and accessory genes located within rearranged gene blocks, relative to their distribution in the full genome set.

Genome rearrangements are thought to be driven by internal processes like homologous recombination and the activity of viral recombinases, transposons, and other MGEs (Achaz et al. 2003; Nakagawa et al. 2003; Brüssow et al. 2004; Rocha 2004, 2006; Toussaint and Rice 2017). To investigate the potential drivers of the genome rearrangements reported in our dataset, we analyzed the function of the core and accessory genes found in the rearranged gene blocks. Although core genes were used to define gene blocks, our analysis also included the accessory genes located between the core genes within the rearranged fragments. We explored the prevalence of different functional categories in translocated and inverted blocks relative to the functional composition of all the genomes in our dataset (See Materials and Methods). We found a similar functional composition between translocations, inversions, nonrearranged regions, and the functional categories found across all the genomes (Fig. 5a–d). To further investigate what specific functions may have a role in genome rearrangements, we identified the Cluster of Ortholog Groups (COGs) statistically enriched in translocations and inversions. Enriched COGs were defined as COGs presenting a Benjamini–Hochberg corrected *P*-value below 0.05 using a *χ*^2^ test that compared their frequency in rearranged blocks relative to their overall expected frequency across the entire genome. Translocations and inversions showed a prevalence of enriched COGs that are involved in carbohydrate transport and metabolism, translation, ribosomal structure and biogenesis, inorganic ion transport and metabolism, signal transduction and metabolism, coenzyme transport and metabolism, transcription, and energy production and conversion (adjusted *P* < 0.05; Table S6). We further focused our analyses on the COGs that belong to the categories “Mobilome: prophages, transposons” and “Replication, recombination and repair” (adjusted *P* < 0.05; Fig. 5) due to their expected role on genome rearrangements. The complete list of the COGs enriched in rearrangements can be found in Table S6.

**Fig. 6.**
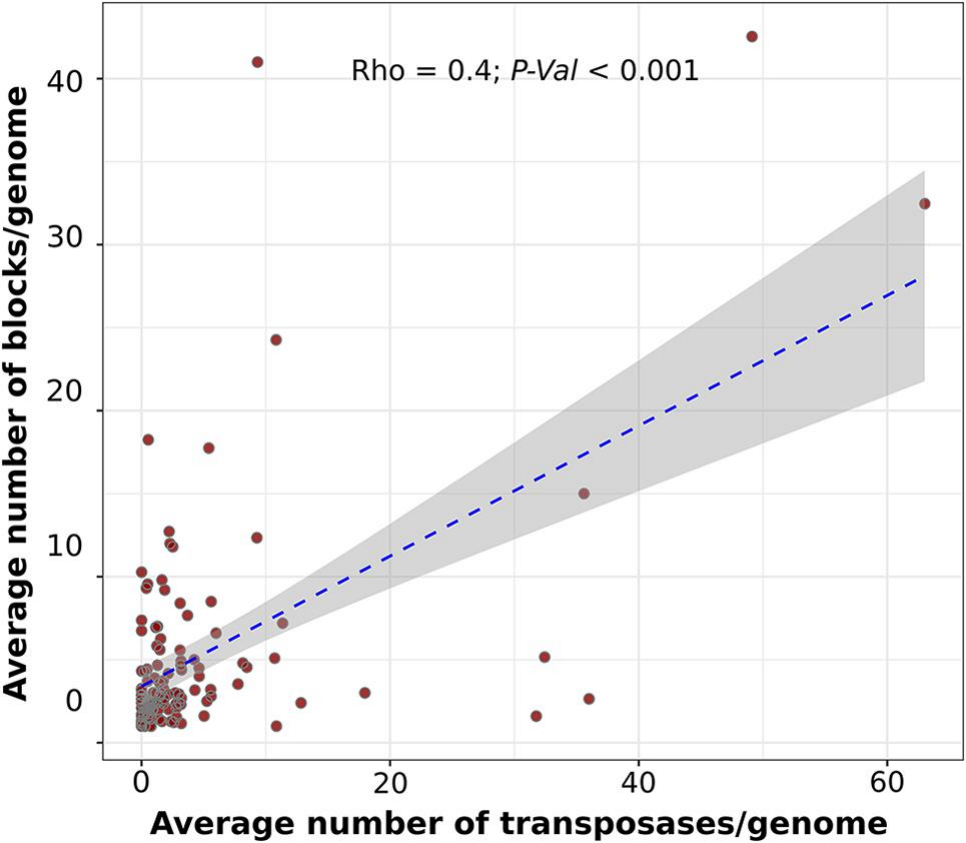
Relationship between the number of blocks found in each genome and the number of proteins annotated as transposases. Each dot represents the average number of blocks and transposases for each species. For each genome, transposase counts were normalized by genome size. Figure S11 indicates that the trend found is maintained despite the exclusion of outliers (>20 blocks).

Overall, our analysis showed that genes involved in the categories “Mobilome: prophages, transposons” and “Replication, recombination and repair” were statistically enriched in the rearranged blocks relative to the content of whole genomes (Table S6). Translocations and inversions showed a similar fraction of genes enriched in these categories. The majority of the enriched core genes in both types of rearrangements were part of the category “Replication, recombination and repair,” such as genes involved in the replication of the chromosome (e.g. DnaA, DnaC, and helicases). We also found an over-representation of genes encoding DNA repair proteins like Endonuclease UvrABC ATPase subunit, Recombinational DNA repair ATPase RecF, and DNA mismatch repair protein MutH, among others. In addition, we found an enrichment of genes annotated as involved in the integration of prophages, genes involved in the duplication, recombination, and repair of DNA, and particularly genes annotated as transposases. We also observed a significant correlation between the total number of transposases and the number of rearranged gene blocks in genomes (Spearman correlation, *ρ* = 0.4; *P* < 0.001; Fig. 6). This suggests that genomes undergoing more rearrangements tend to have a higher number of transposases. This finding is consistent with previous observations indicating that genome instability increases with the expansion of inverted repeats (Achaz et al. 2003). Although we observed a larger number of rearrangements near *Ori*, transposases were not found at a particularly higher occurrence in this region; instead, they were relatively evenly distributed across the genomes with a slight increase near *Ori* (Fig. S10). This suggests that, if transposons indeed play a role in mediating genome rearrangements, either (i) not all rearrangements are mediated by transposases or (ii) transposases are rapidly lost at these sites following a rearrangement. This last interpretation is consistent with the inherently dynamic behavior of transposable elements (Schmitz and Querques 2024).

The accessory genes enriched in rearranged blocks showed a large diversity of COGs in the category “Mobilome: prophages, transposons,” particularly transposases and other transposase- and phage-related genes. Examples of these genes are Transposase IS1182 family, Transposase and inactivated derivatives IS5 family, and Phage terminase large subunit, respectively. To a lesser extent, the accessory genes found in rearranged blocks were enriched in genes involved in the repair of DNA and recombination (Table S6).

## Discussion

In this study, we explored the dynamic and the potential drivers of genomic rearrangements across a broad diversity of bacterial and archaeal species. Our results indicate that, overall, genome rearrangements are rare events within species (Figs. 1 and 2a), which is in agreement with their expected deleterious effect for cell physiology and genome stability (Hendrickson and Lawrence 2006; Esnault et al. 2007). Moreover, our study supports previous findings that point to the maintenance of gene clustering of genes at different timescales (Rocha 2006; Fang et al. 2008; Junier and Rivoire 2013), which we observed here in the core genome of species (Fig. 2a). On average, pairs of genomes from the same species displayed three syntenic blocks, which according to our predictions is typically the result of a single rearrangement (Fig. S12). This finding indicates that the genomes of a given species are largely syntenic and most species display less than two rearrangements. The rare occurrence of rearrangements in our dataset together with previous findings suggests that these events are very rare and/or that they are strongly counter selected when they occur. Similarly, we also found that when rearrangements are observed, they span a large number of genes (Fig. 2b), suggesting that small rearrangements are as deleterious as larger ones. Note that, as opposed to some other studies (Eisen et al. 2000; Tillier and Collins 2000; Suyama and Bork 2001; Belda et al. 2005; Merrikh and Merrikh 2018), our analyses focused on rearrangements that occurred across short evolutionary timescales (i.e. within a species).

Although few arrangements were observed in most species, we found extreme instances at both ends of the spectrum. Some species showed a complete absence of genomic rearrangements, whereas others exhibited evidence of frequent movement of gene blocks across their genomes (Fig. 2c). Among the species where genomic rearrangements were not identified were *B. aphidicola, T. pallidum, M. morganii, Br. aurantiacum, Bo. garinii, Bo. burgdorferi, and Bor. avium.* These species, with the exception of *Br. aurantiacum,* are characterized by having symbiotic lifestyles, which likely limits the gain of MGEs from external sources (as detailed below). Moreover, host-dependent microorganisms like *B. aphidicola* have lost genes involved in DNA repair and recombination (Moran and Mira 2001; Tamas et al. 2002; van Ham et al. 2003), two processes involved in rearrangements through intrachromosomal recombination (Darling et al. 2008). In contrast, species with a large estimated number of rearrangements like *A. salmonicida, Bor. pertussis, T. phagedenis, and X. oryzae,* have been reported to have a high number of transposons and other MGEs (Salzberg et al. 2008; King et al. 2010; Mushtaq et al. 2015; Sealey et al. 2016; Charette 2021). These processes are expected to contribute to the frequent rearrangement events observed in these genomes.

Our study shows that translocations are slightly more prevalent than inversions across the species analyzed in our dataset (Fig. 2d). Although they were rare overall, inversions were found in many of the genomes where rearrangements were detected, a finding that contrasts with their expected deleterious effect on cell fitness (Casjens 1998). The occurrence of inversions in many of the genomes analyzed suggests that these events occasionally have a neutral or minimal effect on cellular fitness. Indeed, it was demonstrated that in *E. coli*, some inversions occurring in the same replichore are only slightly detrimental, regardless of changes in gene orientation (Rocha 2004, 2008; Esnault et al. 2007). Interestingly, we found that not only inversions, but also translocations tend to be enriched near *Ori*, and to a lesser extent *Ter* (Fig. 3a and b). This finding contrasts with a previous study that found a higher incidence of inversions near *Ter* relative to *Ori* (Suyama and Bork 2001).

Our findings indicate that *Ori* has a higher propensity for rearrangements relative to other regions of the genome. Alternatively, a more frequent relocation of genomic blocks near *Ori* may reflect an adaptive advantage to move some genes to this region through the benefit of gene dosage effect (Couturier and Rocha 2006). DNA replication can be initiated multiple times before the first round of replication is completed, which results in multiple copies of the area proximal to *Ori* and therefore an increase in the expression of neighboring genes (Anon 1986; Nielsen et al. 2007; Bochkareva et al. 2018; Noureen et al. 2019). Gene dosage effect may favor the relocation of genes closer to *Ori* to increase gene expression, which may constitute a rapid adaptive path to enhance gene expression. For instance, evidence suggests that changes in the location of genes from the secondary to primary chromosome in *Burkholderia,* a species known for having multiple chromosomes, can enhance the expression of genes and potentially lead to positive effects at the fitness level (Morrow and Cooper 2012). In agreement with this idea, we observed that many of the rearranged blocks were enriched in genes involved in key cellular processes such as translation, transcription and carbohydrate metabolism. The relocation of these genes near *Ori* may confer a rapid adaptive response to certain selective pressures. The rare occurrence of rearrangements at *Ter* relative to *Ori* suggests that when the relocation of genes occurs, these events are more strongly counter selected in this region potentially due to the stronger deleterious effects of polarity changes or the disruption of essential gene structures. Indeed, inversions occurring near *Ter* can lead to replichores of different sizes which can then lead to asynchronous convergence of the replication forks, and cause potential issues for chromosome deconcatenation and segregation (Niki and Hiraga 1998; Rocha 2008). Moreover, *Ter* is the target for proteins that allow the correct segregation of chromosomes after replication (Crozat et al. 2020), and therefore relocation in this region can negatively affect cell division. Overall, our results indicate that the translocation of genes near *Ori* may enhance cellular fitness by increasing the expression of genes, whereas inversions that do not preserve DNA polarity at *Ter* are more likely to be counter selected.

We observed that the relationship between the number of syntenic blocks and evolutionary distances between strains was not as clear as previously reported for more divergent bacteria (Figs. 4a and  S7) (Brilli et al. 2013; Noureen et al. 2019). The low correlation between gene synteny and evolutionary distance is supported by the remarkable differences we observed in the rate of rearrangements across genomes and species (Fig. 4b and c). Our findings suggest that the mechanisms and evolutionary processes involved in the preservation and discontinuity of rearrangements vary across genomes and species. It must be emphasized, again, that, as opposed to previous studies, our analyses were exclusively conducted over short evolutionary timescales (i.e. between strains) during which such events are rare. It is therefore expected that our results present a much higher variance relative to rearrangement studies conducted between species and genera.

We found an enrichment of prophages and transposons in rearranged blocks, which suggests that these MGEs are driving translocations and inversions in microbes (Figs. 5a–c and 6; Table S6). Our findings are in close agreement with previous studies that pointed to the intrachromosomal movement of genome fragments due to the activity of phages and transposons (Nevers and Saedler 1977; Nakagawa et al. 2003; Parkhill et al. 2003; Brüssow et al. 2004; Moura de Sousa and Rocha 2022). For instance, prophages in the genome of the phytopathogen *Xylella fastidiosa* can serve as anchoring points for homologous recombination and lead to major genome rearrangements (Brüssow et al. 2004). Another study demonstrated that recombination between homologous prophage genes can result in large chromosomal rearrangements (Nakagawa et al. 2003). In addition, it has been shown that the proliferation of phage satellites can facilitate rearrangements in the bacterial genome (Moura de Sousa and Rocha 2022). Similar examples have been reported for transposons (Parkhill et al. 2003; Vandecraen et al. 2017; Wu et al. 2022). In fact, the replication cycle of some bacteriophages and other transposable elements is associated with their movement to new sites in the genome, which may cause the rearrangement of adjacent DNA sequences (Nevers and Saedler 1977; Shapiro 1979).

Although we did not find significant differences in the number of blocks across microbial lifestyles (Fig. S13), a trait previously associated with genome stability (Brilli et al. 2013), we found that the rearrangements in some species may have a relationship with their ecology. Symbiotic bacteria that lack multiple DNA repair and recombination genes were among the species that showed an absence of rearrangements. These results suggest that intrinsic cellular processes such as DNA repair and recombination are mechanisms driving genomic rearrangements. In addition, these organisms also tend to display fewer MGEs than their free-living counterparts. The role of internal processes like DNA repair and MGEs in genomic rearrangements is in agreement with the lower incidence of rearrangements found in endosymbionts and host-associated microbes (Tamas et al. 2002; Naito and Pawlowska 2016). Overall, our analyses suggest that genomic translocations and inversions in the microbial species that we analyzed are mostly driven by the movement and proliferation of transposable elements and prophages, and potentially processes involved in recombination and DNA repair.

## Materials and Methods

### Genomic Data Collection and Identification of Genome Rearrangements

In order to assess the prevalence of genome rearrangements in bacteria and archaea, we first compiled a genomic dataset that consisted of all the genomes available in the GTDB (release 207) (Parks et al. 2018, 2020, 2022; Rinke et al. 2021) after quality filtering. Quality control consisted in retaining genomes that were 100% complete and had a contamination estimate below 5% (GTDB dataset; Table S1). We only retained genomes that were fully assembled according to assembly information available on NCBI (Sayers et al. 2022) (final dataset, Table S2). After applying these criteria, we only retained the species with more than five genomes. Our final dataset consisted of 5,238 genomes belonging to 119 bacterial species and two archaeal species.

Genomic rearrangements were detected through the identification of core gene blocks within each species. We built the core genome for each species using *CoreCruncher* (Harris et al. 2021) with the parameters -freq 90 -score 80 (i.e. >90% frequency in the species and >80% identity relative to the pivot genome). Note that *CoreCruncher* is inferring orthologs while explicitly testing for the presence of paralogs and xenologs based on the distributions of sequence identity. Overall, core genome size was strongly correlated with the average genome size of each species (Fig. S9a). Gene blocks were identified using custom Python code (https://github.com/carolinaamg/prok_gene_blocks) and consisted in identifying breakpoints of consecutive core genes. Disrupted blocks were located based on pairwise comparison of the order of core genes in genomes from the same species (query vs. reference genome). Blocks consisting of a single core gene were discarded as these cases could be the result of errors in orthology assignment. To obtain the final number of blocks for each genome, we used a parsimonious approach: we used the reference genome that would lead to the lowest average number of blocks across all the genomes of the same species.

To assess the impact of sequencing technology on gene block estimates, the files containing the assembly statistics for each were retrieved from RefSeq. Genomes were classified as “short-read sequenced” or “long-read sequenced” based on the technology used for their sequencing process. Genomes generated with Illumina, 454, Sanger, Ion Torrent, DNBSEQ, and BGISEQ were classified as “short-read sequenced,” whereas genomes sequenced with PacBio and Nanopore were labeled as “long-read sequenced.” Hybrid assemblies using short-read sequencing and long-read sequencing were classified as “long-read sequenced.” We only compared species that had at least five genomes sequenced with each technology (Fig. S2). Because our analyses showed that genomes generated through short- and long-read sequencing resulted in similar numbers of gene blocks, the genomes assembled from both sequencing technologies were retained for further analyses.

### Identification of the Position of Genome Rearrangements Relative to *Ori* and *Ter*

We identified the Origin (*Ori*) and Terminus (*Ter*) of replication in each genome by computing the cumulative GC skew (*S*) using a sliding window of 1,000 bp with *S* = (G − C)/(G + C) (Grigoriev 1998; Bohlin et al. 2010; Lu and Salzberg 2020). Due to differences in the mutation bias of the leading and lagging strands, *Ori* and *Ter* can be identified as the global minimum and maximum of the cumulative GC-skew of the DNA fragments analyzed, respectively. For this analysis, we excluded species known for having more than one chromosome or linear chromosome. We further excluded the genomes whose GC-skew plots were too ambiguous to clearly identify *Ter* and *Ori* (i.e. multiple peaks and valleys were present). Additionally, genomes with only one gene block were discarded for this analysis since they did not show any occurrence of rearrangements.

We next explored whether the location of genomic rearrangements showed a bias toward *Ori* or/and *Ter*. We identified the location of the middle of each rearrangement block relative to *Ori* and *Ter* using custom Python code. Distance values ranged from 0 to 1 and indicated the relative location from *Ori* and *Ter* for both replichores, respectively. Next, we compared each distance distribution with the expectation under neutrality (average distance of 0.5) through a Wilcoxon signed rank test. We excluded genomes that had only one block (absence of genomic rearrangements) and gene blocks that appeared more than twice in our dataset to avoid counting the same rearrangement events multiple times.

### Estimation of Genome Rearrangement Rates Across Bacterial Lineages

The evolutionary distances between pairs of genomes were calculated using a nucleotide concatenated alignment consisting of the core genome of each species as reconstructed by *CoreCruncher* (see above). Alignments were generated for each core gene individually using MAFFT (Katoh and Standley 2013) with default parameters. Once aligned, core genes were concatenated using custom Python code (https://github.com/carolinaamg/prok_gene_blocks). We used RAxML (Stamatakis 2014) to estimate the evolutionary distance between each of the genome pairs and the corresponding number of gene blocks. Distances were computed with RAxML with the option -f x and the substitution model GTRGAMMA for all species except *Bor. holmessi*, for which we used the model GTRGAMMAI due to the prevalence of invariable sites.

We estimated the rate of genomic rearrangements in our dataset. To generate accurate and comparable estimates of the number of rearrangements across species, we simulated genomic rearrangements and used the resulting number of blocks to fit a quadratic regression (Fig. S12). We next used the equation that resulted from this regression to predict the number of rearrangements from gene block data. Then, we computed the rearrangement rate, denoted by *B_cs_*, as follows:


$$Bcs=\frac{N}{C\cdot S\cdot L}$$

Where *N* is the number of predicted genomic rearrangements, *C* is the number of genes in the core genome, *S* is the number of nucleotide substitutions in core genome between pair genomes, and *L* is the length of core genome concatenated alignment.

In our analysis, *B_cs_* is the number of blocks per substitution normalized by the core genome size and alignment length.

### Phylogenetic Reconstruction

To explore the phylogenetic breadth of the genomes used in our analyses, we reconstructed a phylogenomic tree by using 30 conserved marker genes that encode ribosomal and RNA polymerase subunits through the program MarkerFinder (Martinez-Gutierrez and Aylward 2021). The program pipeline consists in identifying the marker genes using HMMER v3.4 (Eddy 2011) followed by the alignment of the individual amino acid sequences with Clustalo v1.2.4 (Sievers and Higgins 2018) with default parameters and their concatenation. The concatenated alignment was then trimmed with trimAl v1.4.rev15 (Capella-Gutierrez et al. 2009) using -gt 0.1, and the phylogeny was reconstructed with IQ-TREE 1.6.9 with the options -wbt -bb 1000 -m TEST (Minh et al. 2013; Nguyen et al. 2015; Kalyaanamoorthy et al. 2017). The best fitting model LG + I + G4 was used for tree reconstruction. To assess the phylogenetic signal in block data, the species tree generated, and the number of blocks were used as input for the function “phylosig” available in *phytools* (Revell 2012).

### Functional Annotation of Genomes and Enrichment Analysis

In order to gain insights into the potential drivers of genome rearrangements in the genomes in our dataset, we performed a functional annotation using the EggNOG database (v5.0) (Huerta-Cepas et al. 2019) through the hmmsearch tool implemented in HMMER 3.2.1 (Eddy 2011) with an *e*-value threshold of 10^−5^ on all proteins. Proteins with multiple annotations were filtered to keep the best-scored annotation. To find COGs enriched in genome rearrangements relative to all the genomes, we performed a *χ*^2^ test on each COG found in translocations and inversions. The contingency matrix for each analysis consisted in the count for a given COG in rearranged blocks, the counts of other COGs in the rearranged blocks, the count of the given COG in all the genomes, and the count of other COGs in all the genomes. We excluded genomes that were composed of a single block (absence of genomic rearrangements) and blocks that were defined as the backbone of the genome (i.e. the longest block of each genome).

## Supplementary Material

evag002_Supplementary_Data

## Data Availability

All genomes used in this study are publicly available through NCBI and the Genome Taxonomy Database (GTDB). The code used for genome blocks identification and analysis is available on GitHub at https://github.com/carolinaamg/prok_gene_blocks.
