# Supplementary material for: Prevalence and Evolutionary Implications of Genome Rearrangements in Bacteria
Source: Genome Biol Evol. 2026 Jan 23;18(2):evag002. doi: 10.1093/gbe/evag002 (PMC12863080; doi:10.1093/gbe/evag002)
Supplement: evag002_Supplementary_Data [file evag002_supplementary_data.zip › Supplementary_Figures.docx]

**Supplementary Figures**

**
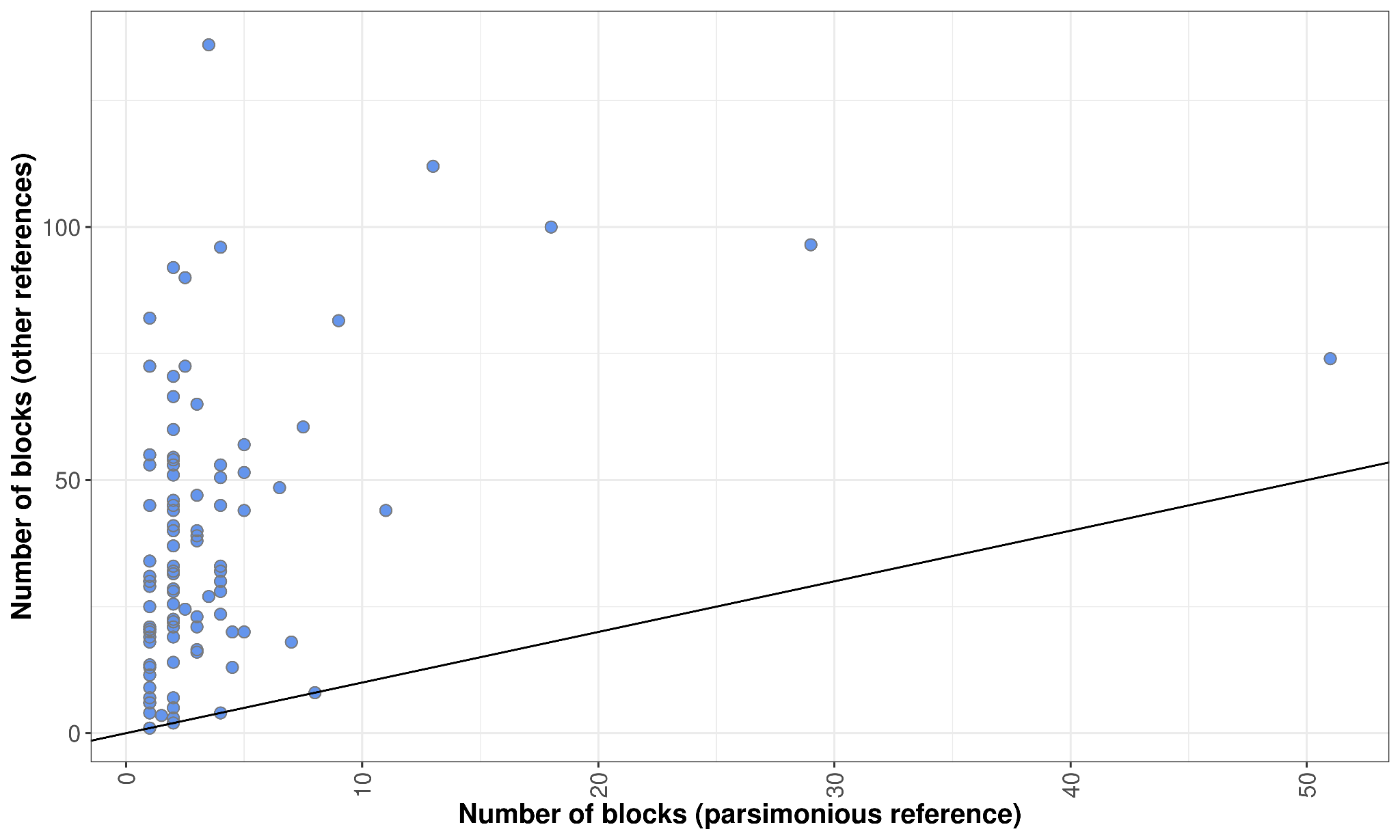
**

**Supplementary Figure 1.** Effect of reference genome choice on the estimated pairwise number of gene blocks. Line indicates a X=Y relationship.


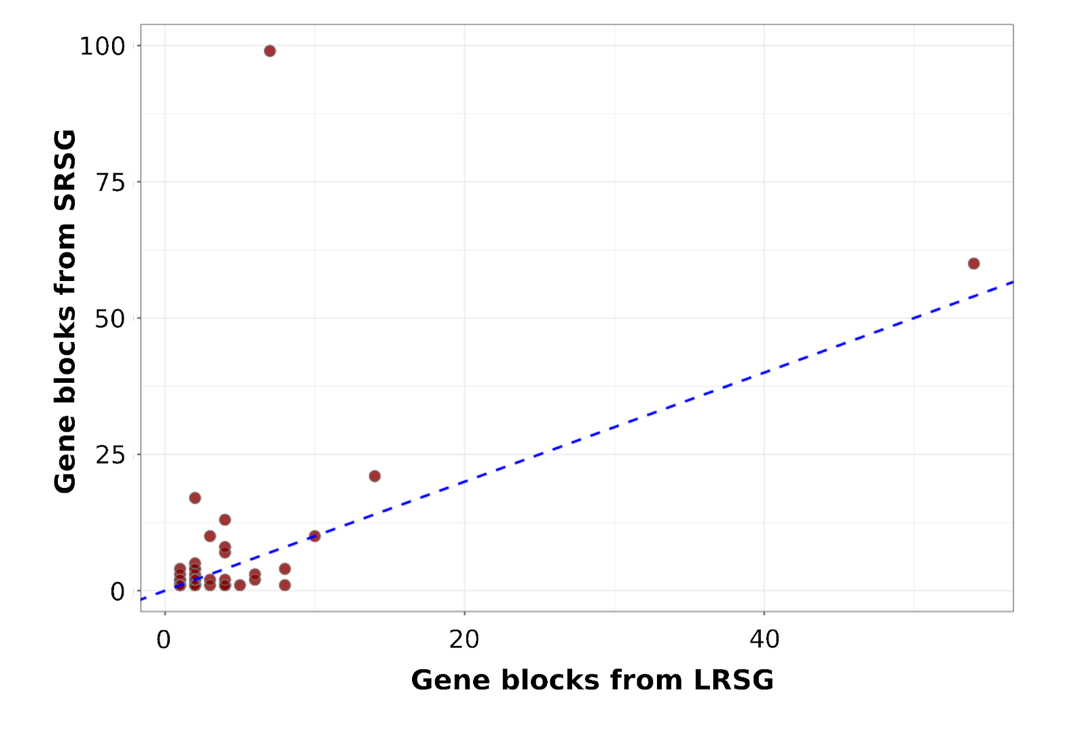


**Supplementary Figure 2.** Comparison of block counts identified from long-read (LRSG) and short-read (SRSG) sequenced genomes, with each dot representing a species.


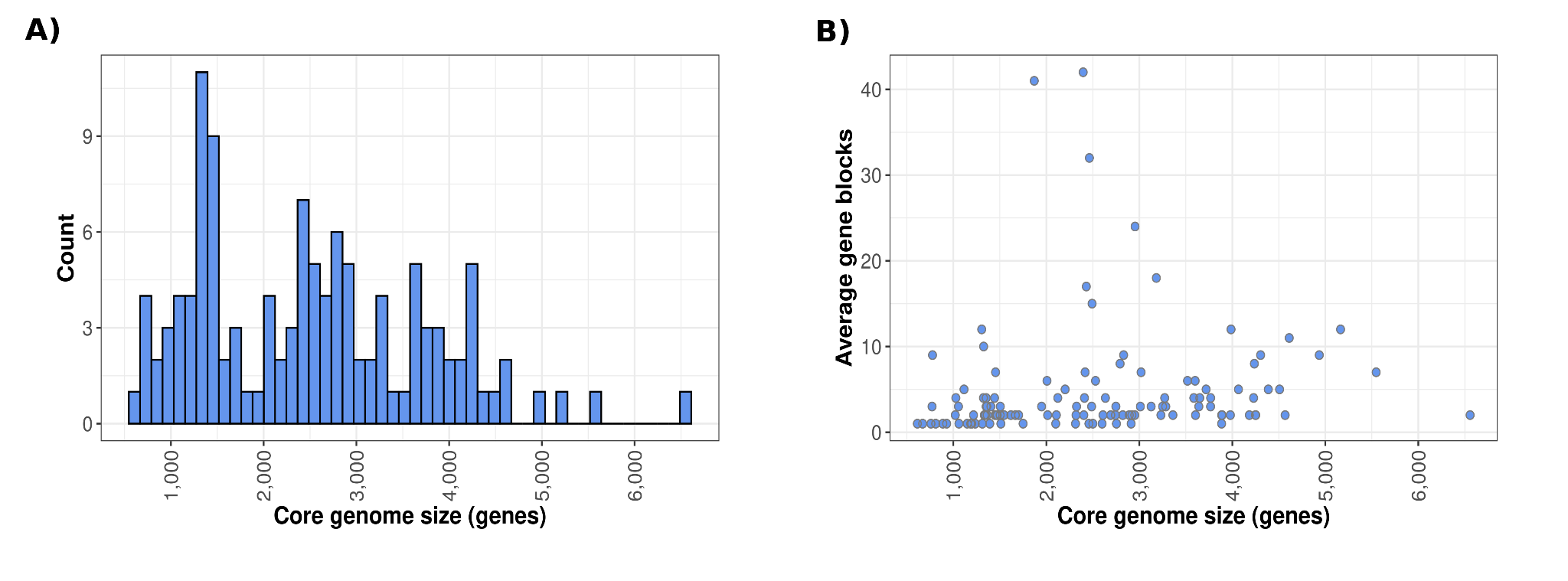


**Supplementary Figure 3.** Distribution of core genome size across the species investigated (A) and relationship between core genome size and the average number of blocks within each species (B).


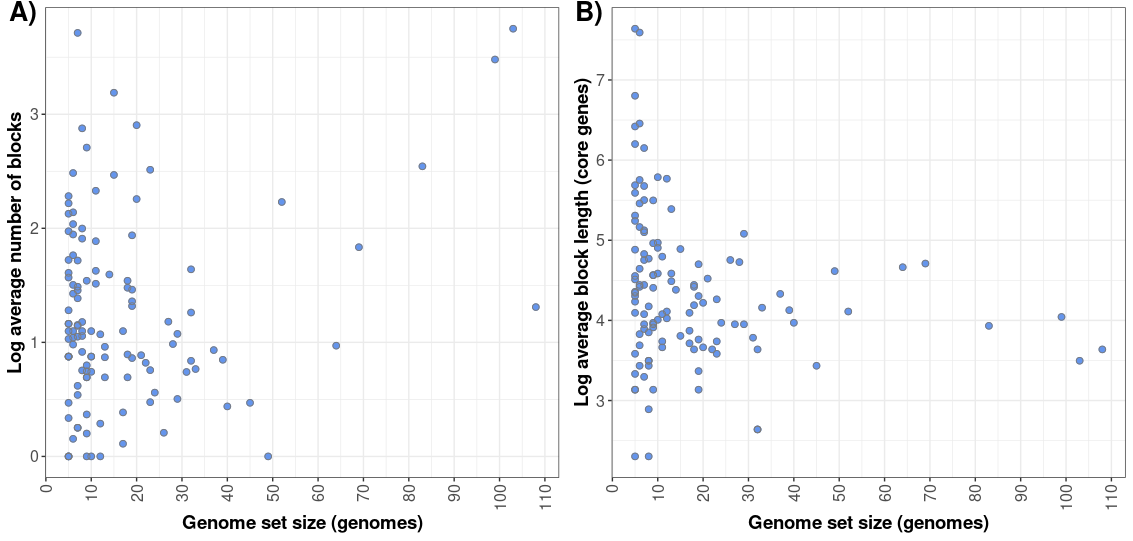


**Supplementary Figure 4.** Relationship between the number of genomes used for each species and the average number of blocks (A) and block length (B).


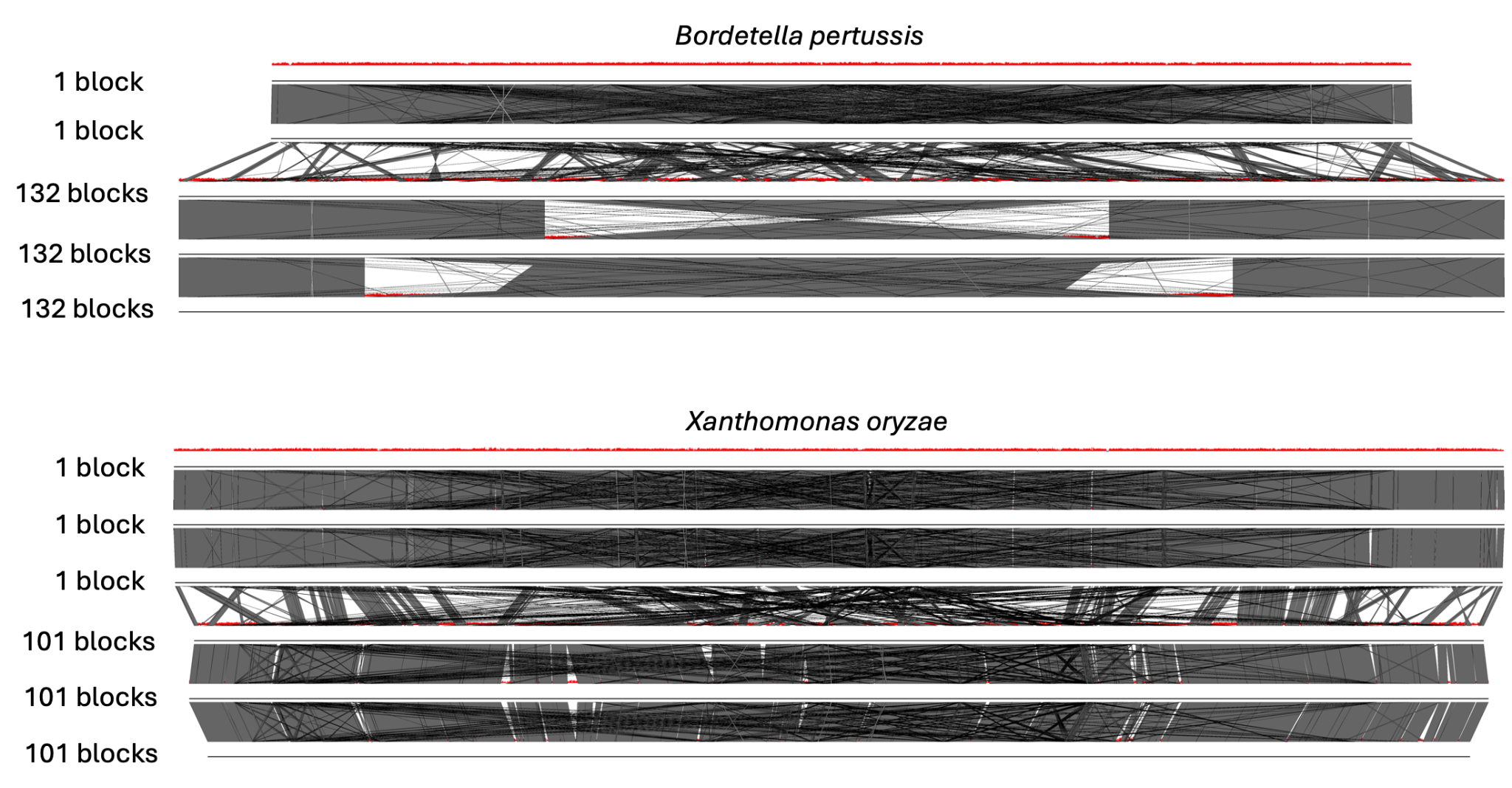


**Supplementary Figure 5.** Synteny of highly fragmented genomes of B. pertussis and *X. oryzae*. Synteny graphs were generated with EasyFig.


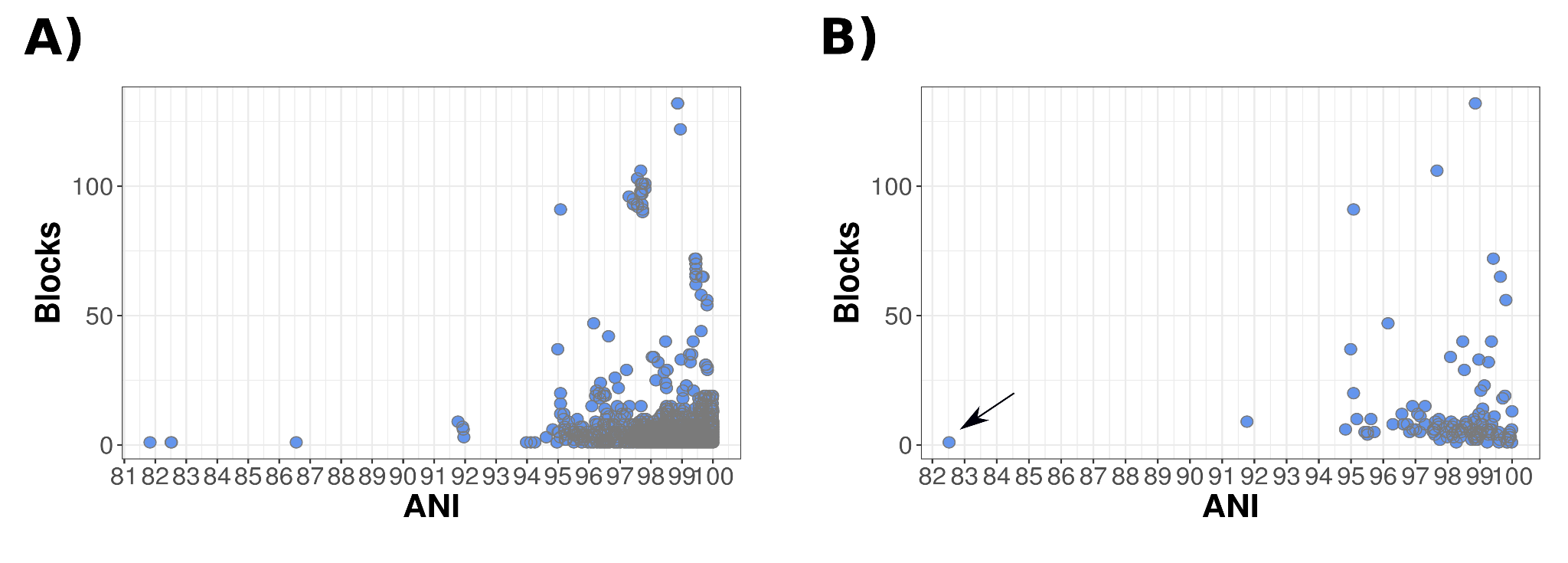


**Supplementary Figure 6.** Relationship between the number of gene blocks and ANI. (A) Pairwise comparisons across the entire genome dataset and (B) comparison between the genome pair with the maximum number of blocks and their corresponding ANI estimate for each species. The black arrow points to *Buchnera aphidicola*, a species with a large divergence between the query and the reference genome while maintaining syntenic genomes.


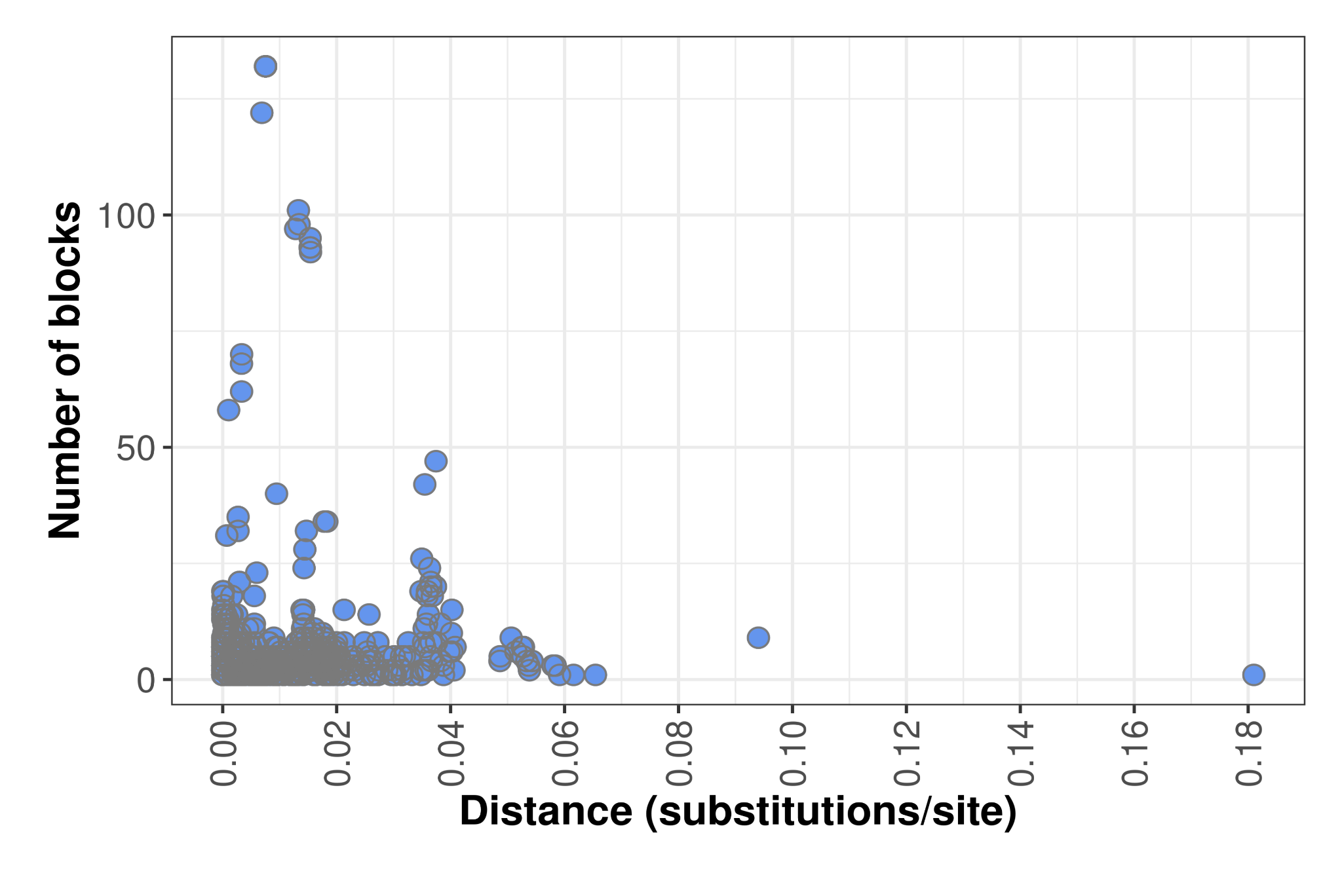


**Supplementary Figure 7.** Relationship between phylogenetic distance and number of blocks.


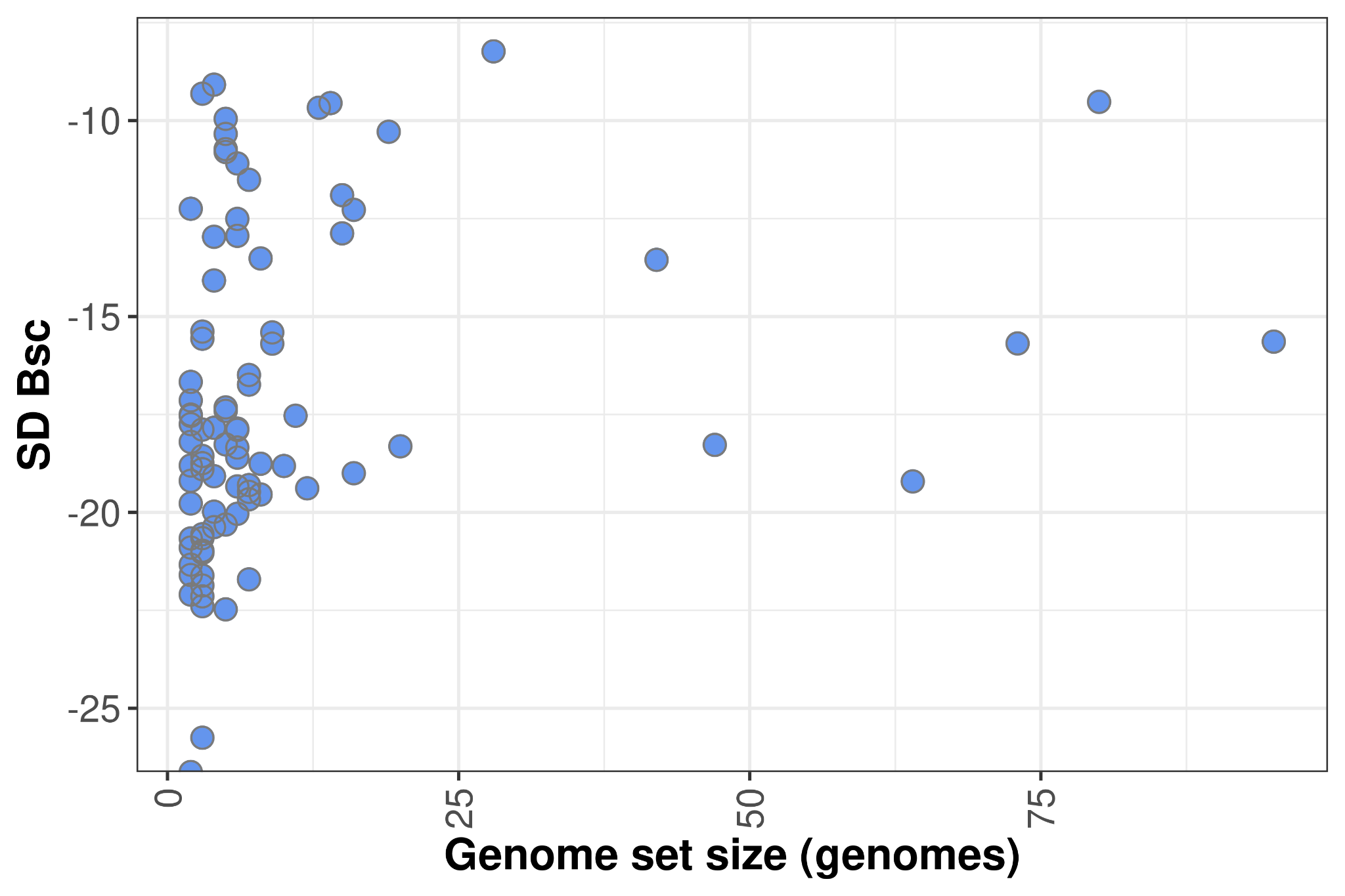


**Supplementary Figure 8.** Relationship between the number of genomes analyzed and the rearrangement rate standard deviation for each species.


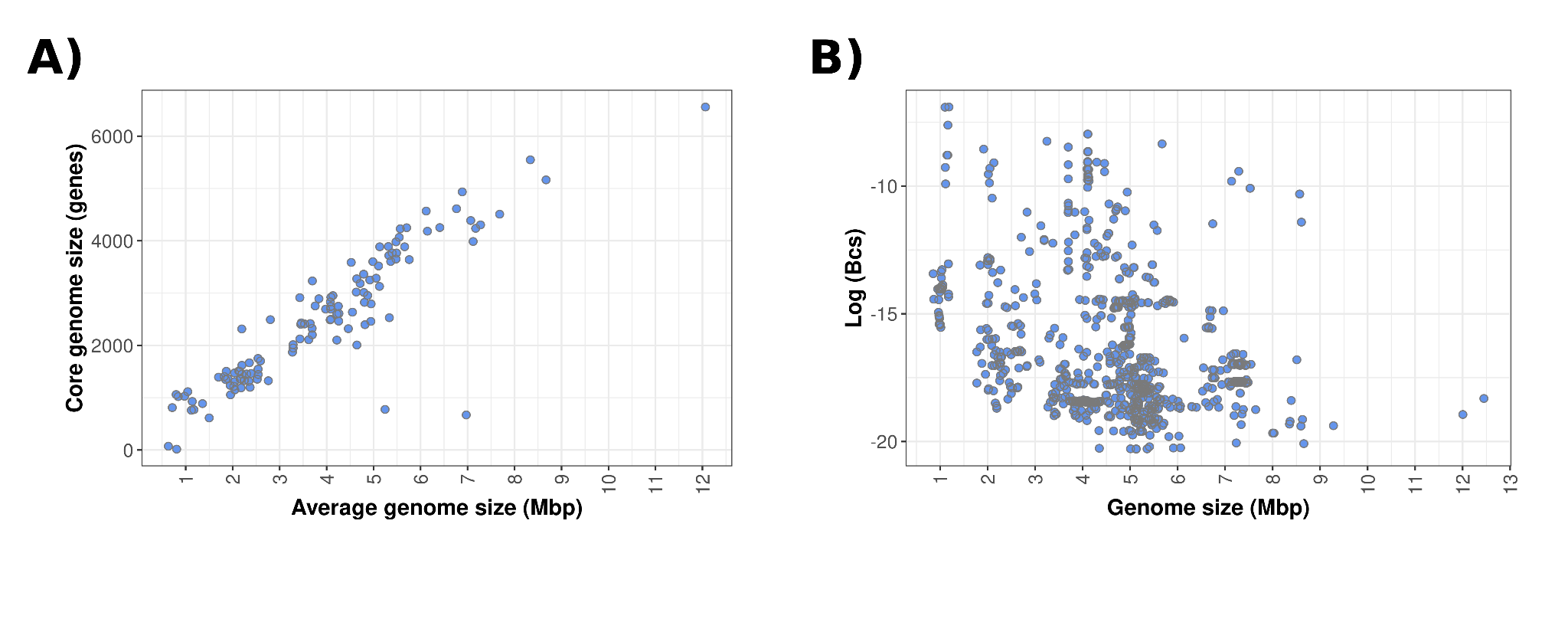


**Supplementary Figure 9.** Relationship between average genome size and core genome size for each species (A) and genome size and rearrangement rate across all genomes (B).


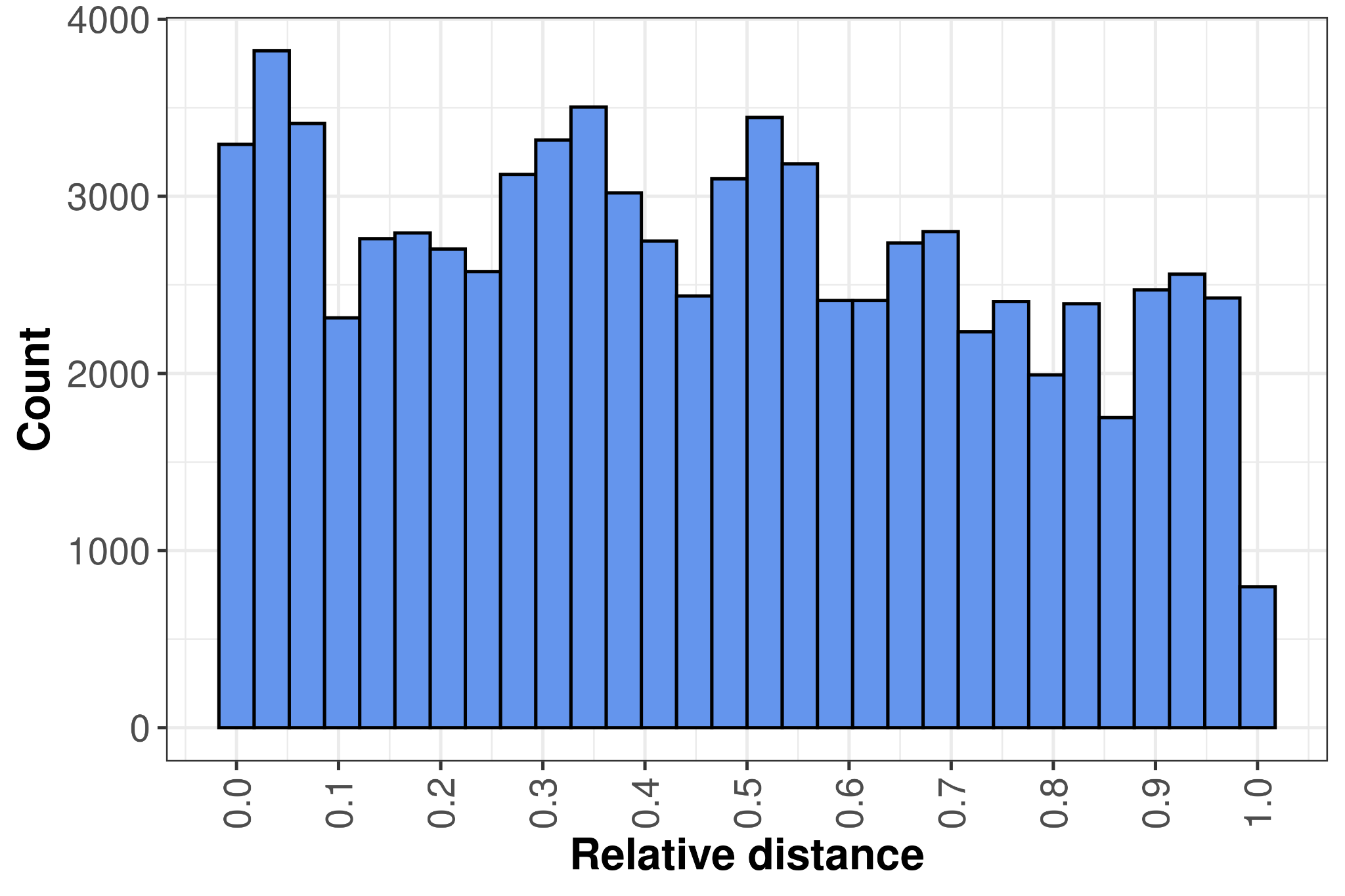


**Supplementary Figure 10.**Relative distance of genes annotated as transposases or transposase-related from the origin of replication (Ori). Values are normalized from 0 to 1, where 0 and 1 indicate close proximity to the Ori.


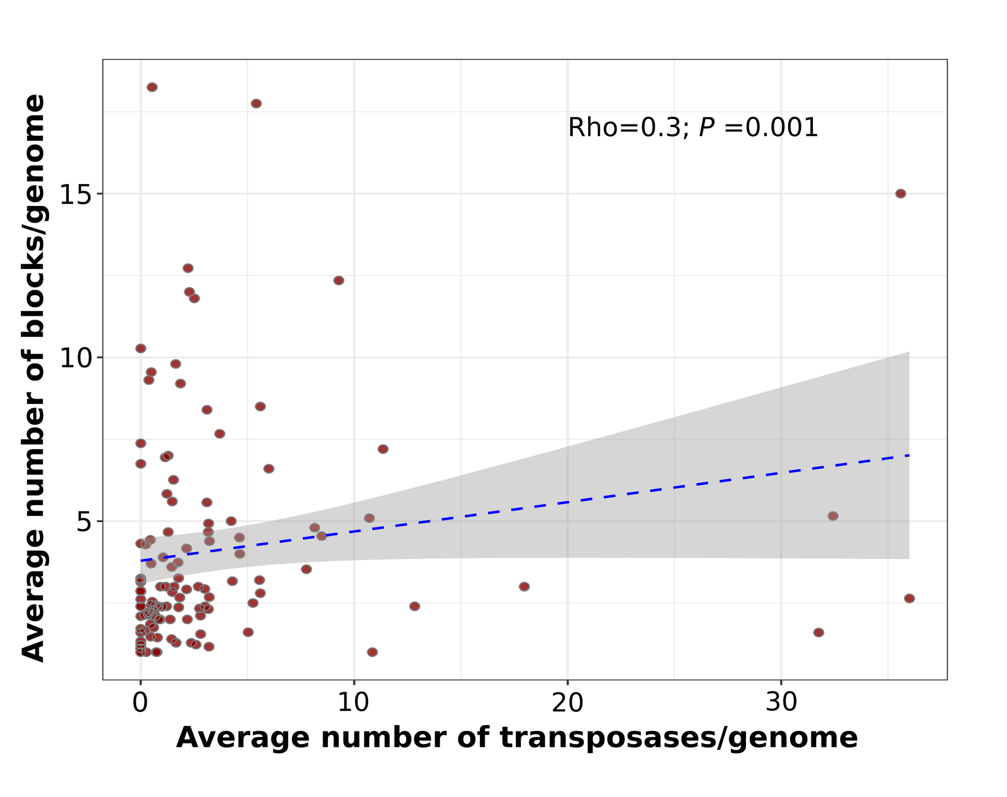
**Supplementary Figure 11.** Relationship between the number of genes annotated as transposases and the number of blocks found in genomes when excluding outlier genomes with >20 blocks.


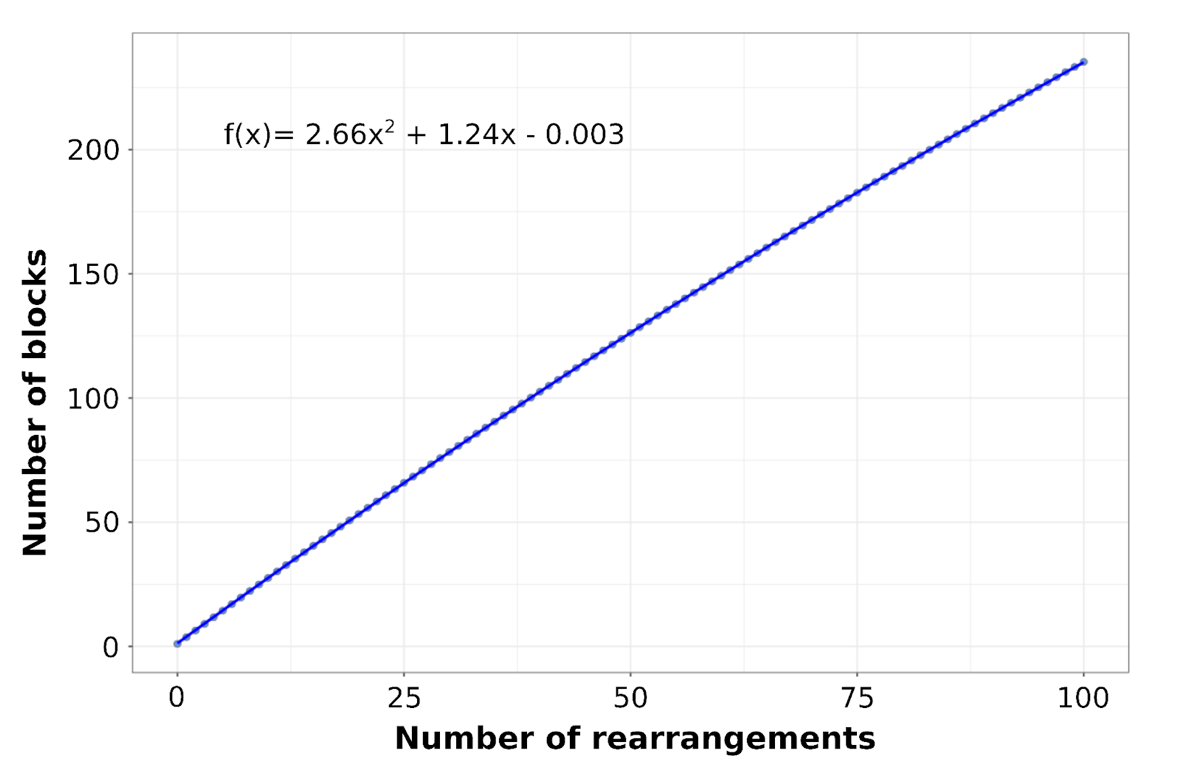


**Supplementary File 12.** Quadratic relationship between the number of predicted blocks that result from simulated rearrangements.


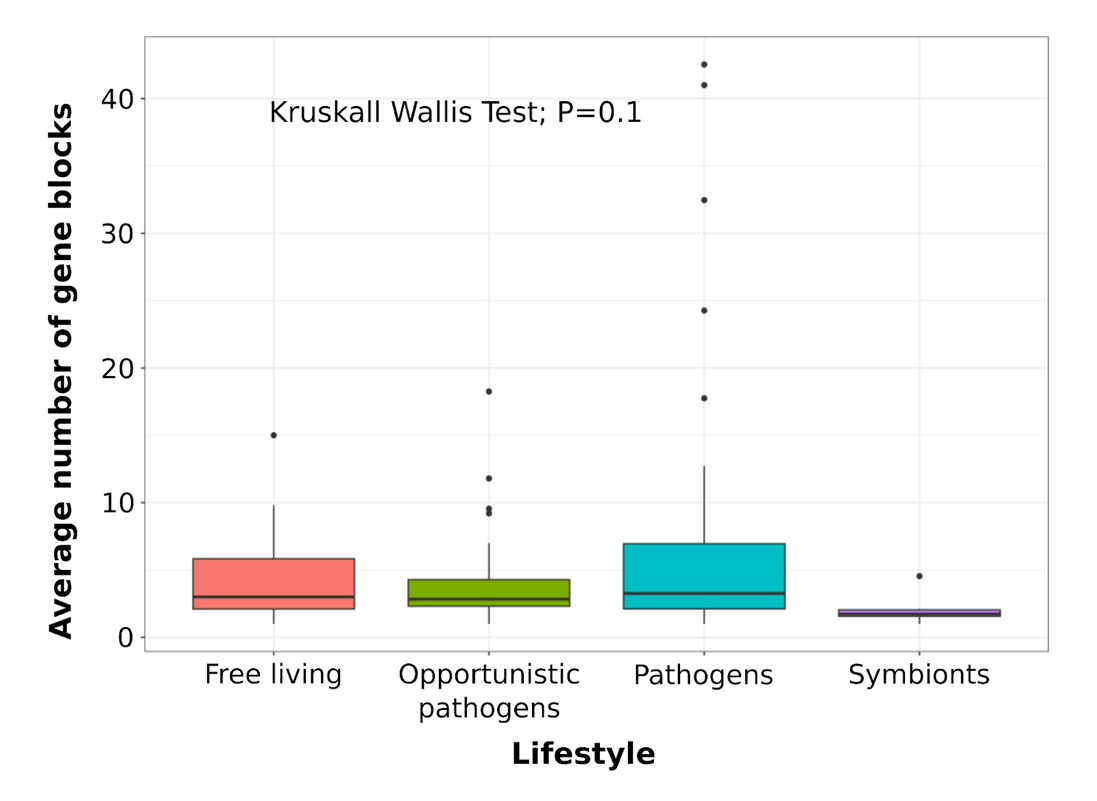


**Supplementary Figure 13.** Number of blocks found in species across different lifestyles.

**SUPPLEMENTARY TABLES**

**Supplementary Table 1.** Genomes retrieved from the Genome Taxonomy Database (GTDB release 207) (Parks et al. 2018; Parks et al. 2020; Rinke et al. 2021; Parks et al. 2022) (GTDB dataset).

**Supplementary Table 2.** Genomes used for gene block identification (Final dataset).

**Supplementary Table 3.** Number of blocks identified in each genome in the final dataset when comparing their core gene order with the reference genome used (See Supplementary Table 5).

**Supplementary Table 4.** Assembly statistics of the genomes used for the estimation of gene blocks, including sequencing technology and assembly method.

**Supplementary Table 5.** Information on the average number of blocks found for each species, the number of genomes used for each species, and the reference genome for each species.

**Supplementary Table 6.** Enriched and non-enriched COGs found in translocations and inversions, including accessory and core genes.
